# Single and double faecal immunochemical test strategies are effective in risk stratification for patients with symptoms of per rectal bleeding suggestive of colorectal cancer

**DOI:** 10.1093/bjsopen/zraf100

**Published:** 2025-10-08

**Authors:** Fatima Shah, Frances Gunn, Malcolm G Dunlop, Edinburgh Colorectal Group, A Clark, M Collie, D Collins, M Duff, S Goodbrand, J Mander, H Paterson, M Potter, C Reddy, D Speake, F Shaban, G Smith, P Vaughan-Shaw, N Ventham, Farhat V N Din, Adam D Gerrard

**Affiliations:** Department of Colorectal Surgery, Western General Hospital, Edinburgh, UK; Interface Triage Office, Western General Hospital, Edinburgh, UK; Cancer Research UK Scotland Centre, Institute of Genetics and Cancer, University of Edinburgh, Edinburgh, UK; UK Colon Cancer Genetics Group, Medical Research Council Human Genetics Unit, Medical Research Council, Institute of Genetics & Cancer, Western General Hospital, University of Edinburgh, Edinburgh, UK; Department of Colorectal Surgery, Western General Hospital, Edinburgh, UK; Cancer Research UK Scotland Centre, Institute of Genetics and Cancer, University of Edinburgh, Edinburgh, UK; Department of Colorectal Surgery, Western General Hospital, Edinburgh, UK; Cancer Research UK Scotland Centre, Institute of Genetics and Cancer, University of Edinburgh, Edinburgh, UK

## Abstract

**Background:**

Faecal immunochemical test (FIT) results triage urgent suspicion of colorectal cancer (USoC) referrals to investigation. As FIT detects microscopic blood, its role in patients with per rectal bleeding (PRB) is controversial. Patients are encouraged to submit sample stools without evident bleeding. The positivity rate, colorectal cancer (CRC) detection accuracy, and benefits from repeated FITs in patients with rectal bleeding are unknown.

**Methods:**

A prospective dataset of USoC referrals for CRC was interrogated for referral symptoms, FIT results, and colorectal investigation outcomes. These were linked to South-East Scotland Cancer Network data to ensure complete CRC outcome data. A FIT result of 10 µg Hb/g or more was considered positive. The primary outcome of interest was diagnostic performance of FIT in patients with PRB compared with symptoms excluding PRB, including sensitivity, specificity, and negative predictive value (NPV). Secondarily, the impact of double FITs in these cohorts was investigated.

**Results:**

A total of 5686 patients completed a FIT and subsequent colorectal investigation, and 2130 (37.5%) of these had PRB as a referral symptom. FIT positivity was higher in patients with PRB compared with no PRB (34.7% *versus* 18.6%; *P* < 0.001). When two successive FITs were completed, the positivity rate rose to 43.5%. Significant bowel pathology (CRC, advanced adenoma, inflammatory bowel disease (IBD)) was more prevalent in patients with PRB. The majority of CRCs in the PRB group were located distally (PRB 94.1% *versus* no PRB 51.5%; *P* < 0.001). The sensitivity for CRC was significantly greater in those with PRB compared with no PRB (98.0% (95% confidence interval (c.i.) 95.1–99.2) *versus* 82.5% (95% c.i. 74.6–88.9)), with respective NPVs of 99.8% and 99.4%. Double FITs increased CRC sensitivity in the non-PRB group, removing the difference in sensitivity between the two groups observed with one test (PRB 100% (95% c.i. 92.3–100) *versus* no PRB 92.9% (95% c.i. 79.4–97.8)). The NPV for CRC in PRB when two FITs were complete was 100% (99.0–100).

**Conclusion:**

Rectal bleeding makes up one-third of USoC referrals to secondary care. The FIT positivity rate is 34.7% and it has a high sensitivity for CRC. Patients with PRB with two negative FITs have a negligible CRC prevalence.

## Teaser

Faecal immunochemical testing (FIT) is effective in detecting colorectal cancer (CRC), even in patients with per rectal bleeding (PRB), a symptom traditionally excluded from FIT use.

Patients with PRB showed higher FIT positivity and greater sensitivity for CRC detection, particularly with a single FIT; no CRCs were missed when two FITs were both negative.

The study supports using double FIT strategies in patients with PRB to safely guide investigation urgency and potentially reduce unnecessary invasive procedures.

## Introduction

Per rectal bleeding (PRB) is a recognized ‘high-risk’ symptom of possible colorectal cancer (CRC)^1,2^. However, PRB is a commonly experienced problem, with as many as 20% of patients attending general practice reporting an incidence of PRB within the past 12 months, and often associated with benign anorectal conditions^3^. The faecal immunochemical test (FIT), using single and double FITs, has been shown to aid risk stratification and prioritization of patients with symptoms suspicious of CRC and is now regarded as a first-line investigation^4–6^.

FIT works by detecting microscopic levels of human haemoglobin in faeces. As such, it was initially not recommended for patients with PRB^7^. However, seldom do patients with PRB bleed on every bowel motion, and the FIT kit instructions state that the test should be performed in the absence of frank visible blood. The authors have previously demonstrated the diagnostic performance of single and double FITs in the population referred from primary to secondary care with symptoms suspicious of CRC, including PRB.

This study aimed to compare the positivity rates and diagnostic performance of a single and/or double FIT strategy for patients with a symptom of PRB compared with no PRB.

## Methods

### Study population and testing

**Fig. 1:**
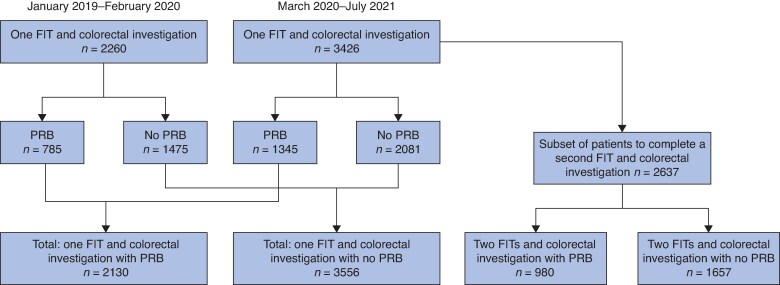
Study flow diagram FIT, faecal immunochemical test; PRB, per rectal bleeding.

This study formed a subset analysis of the authors’ previously published FIT study^6^. In short, all patients referred from primary care with symptoms suspicious of CRC were sent a single FIT (January 2019–February 2020) or two sequential FITs a median of 13 days apart (March 2020–July 2021) (*Fig. 1*). Patients were investigated regardless of FIT return and results.

FIT kits (Minaris Medical Co., Ltd, Tokyo, Japan) were mailed to patients with instructions on how to perform the test. Completed tests were processed at the UKAS-accredited NHS Tayside Blood Sciences laboratory based in Ninewells Hospital (Dundee) with samples analysed to ISO15189 standards using the HM-JACKarc^TM^ analyser (Minaris Medical Co., Ltd, Tokyo, Japan). The test positivity threshold was faecal haemoglobin at 10 μg Hb/g.

The symptoms for referral to secondary care for all patients were prospectively entered into a database. Biochemical, radiological, and endoscopic outcome data were then reviewed. Registry CRC data follow-up was cross-checked to ensure complete capture of cancers. For this analysis, any patients with a symptom of PRB with or without other symptoms were in the PRB group and compared with the no PRB group. Anaemia was defined as a serum haemoglobin level less than 135 g/l in men or 120 g/l in women, in accordance with the local laboratory reference range. Significant bowel pathology (SBP) included CRC, advanced adenomas, and IBD.

As this work formed part of standard routine clinical care, ethical approval was not required. The study was approved by the Western General Hospital Colorectal research and audit committee and was granted local Caldicott approval for the use of patient identifiable data across NHS Lothian (ID#23115).

### Outcomes of interest

The primary outcome was to assess the diagnostic performance of FIT in the subset of patients referred to secondary care along the suspected CRC pathway with symptoms of PRB. This was then compared with the cohort of patients with symptoms other than PRB (no PRB). Secondary outcomes were to assess the utility of a second FIT in a double FIT strategy between the PRB and no PRB groups.

### Statistics

Data analysis was performed using R v4.0.5 (R Foundation for Statistical Computing, Vienna, Austria) with associated packages and GraphPad Prism^TM^ Version 10.3.1 (GraphPad Software, San Diego, CA, USA). Diagnostic accuracies for positive predictive value (PPV), negative predictive value (NPV), sensitivity, and specificity were calculated with 95% confidence intervals (c.i.). The Scottish Index of Multiple Deprivation (SIMD) tool was used to assess levels of socioeconomic deprivation; this creates quintile ranks from the most (1) to least (5) deprived areas from postcodes. Categorical data were compared using the Fisher’s exact test or χ^2^ test, as appropriate, and continuous data were compared using the Student’s *t*-test or Mann–Whitney *U* test.

## Results

### Demographics of patients with PRB

Following referral to secondary care with symptoms suspicious for lower gastrointestinal malignancy, 5686 patients completed a FIT test and subsequent investigation of the colorectum. A subset of 2637 (46.4%) patients completed two FIT tests. In 2130 of 5686 (37.5%) of referrals, PRB was a contributing symptom, and the proportion of patients with PRB was similar in those who completed two tests (*P* = 0.808).

When compared with the patients with no PRB, patients with PRB were more likely to be male, younger, and from the most socioeconomically deprived areas (*Table 1*). Those with PRB were less likely to be taking anticoagulation or antiplatelet medication, or be anaemic (*P* < 0.001).

**Table 1:** Demographics of study population by presence or absence of PRB

	PRB	No PRB	P*
No. of patients	2130	3556	
**Sex**			
Female	1168 (54.8%)	2054 (57.8%)	0.033
Male	962 (45.2%)	1502 (42.2%)	
Median age (i.q.r.)	61 (52–71)	67 (58–75)	< 0.001
**SIMD**			
5	690 (32.4%)	1306 (36.7%)	
4	389 (18.3%)	687 (19.3%)	
3	373 (17.5%)	605 (17.0%)	
2	447 (21.0%)	646 (18.2%)	
1	217 (10.2%)	292 (8.2%)	< 0.001
Anticoagulation	368 (17.3%)	769 (21.6%)	< 0.001
Anaemia	363 (17.0%)	819 (23.0%)	< 0.001

Values are *n* (%) unless otherwise stated. PRB, per rectal bleeding; i.q.r., interquartile range; SIMD, Scottish Index of Multiple Deprivation (5, least deprived). SIMD not available for 1.3%. *χ^2^ test.

### Diagnostic performance of FIT in PRB

The positivity rate of FIT, at 10 µg Hb/g, was greater in patients with PRB than in those with no PRB (34.7% *versus* 18.6%; *P* < 0.001). There was also a greater prevalence of significant bowel pathology (CRC, advanced adenoma, and IBD) in patients with PRB (*Table 2*). Sensitivity for detecting CRC at a 10 µg Hb/g threshold in those with PRB was 98.0% (95% c.i. 95.1–99.2), greater than in those with no PRB at 82.5% (95% c.i. 74.6–88.9) (*Table 3*). The total number of patients that needed to be investigated to diagnose 1 case of CRC in patients with PRB and a FIT less than 10 µg Hb/g was 695. Similarly, sensitivity for detecting significant bowel pathology was greater in patients with PRB. Data for advanced adenoma and IBD can be found in *Table S1*.

**Table 2:** Positivity rate and prevalence of pathology in PRB *versus* no PRB

	PRB	No PRB	P*
No. of patients	2130	3556	
Positivity	740 (34.7%)	663 (18.6%)	< 0.001
CRC	101 (4.7%)	103 (2.9%)	0.004
AA	114 (5.4%)	126 (3.5%)	0.001
ACRN	215 (10.1%)	229 (6.4%)	< 0.001
IBD	80 (3.8%)	33 (0.9%)	< 0.001
SBP	295 (13.8%)	262 (7.4%)	< 0.001

Values are *n* (%) unless otherwise stated. PRB, per rectal bleeding; CRC, colorectal cancer; AA, advanced adenoma; ACRN, advanced colorectal neoplasia; IBD, inflammatory bowel disease; SBP, significant bowel pathology. *χ^2^ test.

**Table 3:** Diagnostic test performance of FIT in PRB *versus* no PRB for CRC and SBP

	Sensitivity	Specificity	PPV	NPV
**CRC**				
PRB	98.0 (95.1–99.2)	68.3 (65.2–71.2)	13.4 (10.6–16.6)	99.8 (99.3–100)
No PRB	82.5 (74.6–88.9)	83.4 (82.3–84.4)	12.8 (9.7–16.7)	99.4 (98.9–99.7)
**SBP**				
PRB	82.1 (73.3–89.3)	72.7 (71.5–73.9)	32.7 (27.2–38.6)	96.2 (94.9–97.2)
No PRB	62.2 (55.1–68.6)	84.8 (83.8–85.7)	24.6 (20.7–29.0)	96.7 (96.0–97.3)

Values with 95% c.i. FIT, faecal immunochemical test; PPV, positive predictive value; NPV, negative predictive value; CRC, colorectal cancer; SBP, significant bowel pathology; PRB, per rectal bleeding.

There were 101 patients with CRC who had PRB, and 103 patients with CRC who had no PRB. A total of 94.1% of the CRCs in the PRB group were distal cancers (splenic flexure to rectum) compared with 51.5% in the no PRB group (*P* < 0.001).

### Use of double FIT in PRB

A subgroup analysis was performed on the 2637 patients who completed two FITs, median 13 days apart, before investigation. The number of patients with PRB was 980 (37.2%), similar to the entire cohort (*P* = 0.808). Again, the prevalence of CRC and SBP was greater in patients with PRB compared with the no PRB group (4.7% *versus* 2.5%; *P* = 0.003, and 12.0% *versus* 6.0%; *P* < 0.001, respectively). The positivity rate where FIT was 10 µg Hb/g or greater was 43.5% in patients with PRB compared with 23.7% in the no PRB group (*P* < 0.001).

The levels of discordance between the repeated FIT results were greater in patients with PRB (21.9% *versus* 13.7%; *P* < 0.001) (*Table 4*). There were no patients with CRC and PRB who had two FITs < 10 µg Hb/g. In patients with PRB where both tests ≥ 10 µg Hb/g, the prevalence of CRC was 20%, meaning only five patients would need to be investigated to diagnose a CRC. The use of double FITs in the no PRB patients improved sensitivity for CRC detection so that, unlike in the single test, sensitivity is non-inferior to PRB (*Table 5*).

**Table 4:** Double FIT strategy in patients with PRB or no PRB

	Double FIT result (µg Hb/g)	Number of patients	CRC (prevalence)	ACRN (prevalence)	SBP (prevalence)
PRB	< 10, < 10	554 (56.5%)	0 (0.0%)	11 (2.0%)	11 (2.0%)
	< 10, ≥ 10	84 (8.6%)	1 (1.2%)	6 (7.1%)	7 (8.3%)
	≥ 10, < 10	131 (13.4%)	3 (2.3%)	13 (9.9%)	13 (9.9%)
	≥ 10, ≥ 10	211 (21.5%)	42 (19.9%)	63 (29.9%)	87 (41.2%)
No PRB	<10 , < 10	1264 (76.3%)	3 (0.2%)	23 (1.8%)	26 (2.1%)
	<10 , ≥ 10	108 (6.5%)	2 (1.9%)	8 (7.4%)	9 (8.3%)
	≥ 10, < 10	119 (7.2%)	4 (3.4%)	11 (9.2%)	11 (9.2%)
	≥ 10, ≥ 10	166 (10.0%)	33 (19.9%)	50 (30.1%)	54 (32.5%)

FIT, faecal immunochemical test; CRC, colorectal cancer; ACRN, advanced colorectal neoplasia; SBP, significant bowel pathology; PRB, per rectal bleeding.

**Table 5:** Diagnostic performance of double FIT strategy for CRC

	Sensitivity	Specificity	PPV	NPV
PRB	100 (92.3–100)	45.6 (42.4–48.8)	8.3 (6.2–10.9)	100 (99.0–100)
No PRB	92.9 (79.4–97.8)	75.7 (73.5–77.8)	9.0 (6.7–12.0)	99.8 (99.3–100)

Values with 95% c.i. FIT, faecal immunochemical test; PPV, positive predictive value; NPV, negative predictive value; PRB, per rectal bleeding.

## Discussion

PRB is a common referral symptom from primary care, contributing to 37.5% of all referrals. SBP is more prevalent in those with a symptom of PRB compared with the absence. Despite concerns of its applicability in patients with PRB, FIT positivity was, as anticipated, higher in patients with overt PRB but certainly not 100% (34.7% for a single FIT, 43.5% for double FITs).

Whilst patients with PRB had a greater prevalence of SBP, they were also more likely to be male and younger in age. PRB as a symptom is frequent in self-limiting benign anorectal disease and significant bowel pathology, making clinical assessment from symptoms alone challenging. FIT allows stratification of this risk for SBP. However, it is also known that PRB patients who are younger and/or male are less likely to engage in FIT testing^8,9^. Therefore, it is imperative that FIT pathways both encourage and support the uptake of testing in these cohorts, and build in mechanisms to assess the FIT non-returners. The reduced number of patients on anticoagulation in the PRB group is likely owing to the group consisting of younger patients who are less likely to be on anticoagulation medication.

The sensitivity of FIT for CRC and SBP was greater in patients with PRB. The uplift in sensitivity seen by adopting a double FIT strategy was only seen in the no PRB group, mainly due to the very high sensitivity of PRB to a single FIT. This gives great confidence in triaging patients with PRB and two FITs less than 10 µg Hb/g to a watch-and-wait policy rather than direct to urgent invasive investigation. The increased sensitivity of FIT in the PRB group is likely multifactorial. Firstly, the positivity rate is higher, not unexpectedly, as FIT is a test of haemoglobin. It is also the case that not all cancers bleed or bleed intermittently, a reason for the false negative FIT result and ‘missed’ CRC diagnosis^10^. Given the referral symptom was PRB, it would stand to reason that it would be less likely that these CRCs were not actively bleeding and therefore were detected by FIT. The majority of the CRCs in the PRB group were also left-sided, distal cancers, which are again known to have a lower miss rate and greater sensitivity with FIT^11^.

The prevalence of PRB in the referred population within this study was comparable to the literature^12,13^ when FIT has been used in symptomatic patients (37.5% *versus* 32.0%^12^  *versus* 31.9%^13^). Consistent with this study, a symptom of PRB was associated with an increased prevalence of CRC and SBP. Compared with the subset analysis of the NICE FIT study^12^, single FIT positivity at a 10 µg Hb/g threshold was greater in this study (34.7% *versus* 26.9%; *P* < 0.001), despite there being a lower prevalence of SBP within the populations with PRB compared with no PRB (13.8% *versus* 17.3%; *P* < 0.001). The increased positivity may have resulted from an increase in benign conditions that can cause PRB within this study population, although this was not a measured outcome from this study, or a consequence of the difference in patient cohorts being studied. Sensitivity data of a positive FIT at 10 µg Hb/g for detecting CRC were comparable with the sensitivity in patients with PRB of 96.6% (95% c.i. 92.2–98.9) reported within the NICE FIT sub analysis, again confirming the increased sensitivity for CRC detection in patients with PRB compared with no PRB (96.6% *versus* 86.3%).

Patients with PRB were more likely to be from the most socioeconomically deprived areas (SIMD 1) than those without PRB. The SIMD distribution of the referrals included in this study were representative of the regional population as a whole (*Table S2*). It is well evidenced that patients from lower socioeconomic areas have a disparity in terms of CRC diagnosis and overall survival^14^.

There are limitations to this study. Firstly whilst large with regards to the number of patients, it is single-centred. The study was not able to report on the duration or severity of symptoms of PRB or the combination of PRB with other traditional higher-risk symptoms. Whilst every patient undertook investigation of the colorectum, not all received a colonoscopy, and therefore assessment of other benign diagnoses for PRB could not be made.

Whilst the positivity rate with two tests is higher, there were no missed patients with CRC. The utilization of FIT testing in PRB is beneficial as it risk stratifies patients with a common referral symptom. When FIT was less than 10 µg Hb/g, the CRC prevalence was 0.18%, and when two tests were both below 10 µg Hb/g, this study did not find any CRC. A double FIT strategy in patients with PRB can be utilized to direct invasive investigations and/or offer a watch-and-wait approach.

## Collaborators

A. Clark (Western General Hospital, Edinburgh, UK); M. Collie (Western General Hospital, Edinburgh, UK); D. Collins (Western General Hospital, Edinburgh, UK); M. Duff (Western General Hospital, Edinburgh, UK); S. Goodbrand (Western General Hospital, Edinburgh, UK); J. Mander (Western General Hospital, Edinburgh, UK); H. Paterson (Western General Hospital, Edinburgh, UK); M. Potter (Western General Hospital, Edinburgh, UK); C. Reddy (Western General Hospital, Edinburgh, UK); D. Speake (Western General Hospital, Edinburgh, UK); F. Shaban (Western General Hospital, Edinburgh, UK); G. Smith (Western General Hospital, Edinburgh, UK); P. Vaughan-Shaw (Western General Hospital, Edinburgh, UK); N. Ventham (Western General Hospital, Edinburgh, UK).

## Supplementary Material

zraf100_Supplementary_Data

## Data Availability

Summary-level data are available on request.
