# Supplementary material for: Single and double faecal immunochemical test strategies are effective in risk stratification for patients with symptoms of per rectal bleeding suggestive of colorectal cancer
Source: BJS Open. 2025 Oct 8;9(5):zraf100. doi: 10.1093/bjsopen/zraf100 (PMC12507080; doi:10.1093/bjsopen/zraf100)
Supplement: zraf100_Supplementary_Data [file zraf100_supplementary_data.docx]

**Single and Double FIT strategies are effective in risk stratification for patients with symptoms of per rectal bleeding suspected of colorectal cancer**

Shah F^1^, Gunn F^2^, Dunlop M.G^1,3,4^, Edinburgh Colorectal Group^1^, Din F.V.N^1,3^, Gerrard A.D^1,3^

^1^ Department of Colorectal Surgery, Western General Hospital, Edinburgh, Scotland, UK

^2^ Interface Triage Office, Western General Hospital, Edinburgh, Scotland, UK

^3.^ Cancer Research UK Scotland Centre, Institute of Genetics and Cancer, University of Edinburgh, Edinburgh, UK

^4.^ UK Colon Cancer Genetics Group, Medical Research Council Human Genetics Unit, Medical Research Council Institute of Genetics & Cancer, Western General Hospital, The University of Edinburgh, Edinburgh, UK

**Corresponding author:**

Farhat Din

Bowel Cancer UK-Royal College of Surgeons of Edinburgh Chair of Colorectal Cancer Research

Cancer Research UK Scotland Centre, Institute of Genetics and Cancer, University of Edinburgh, Crewe Road, Edinburgh, UK, EH4 2XU

[Farhat.Din@ed.ac.uk](mailto:Farhat.Din@ed.ac.uk)

**Supplementary Materials - Index**

| **Supplementary Result Tables** |  |
| --- | --- |
| **Supplementary Table 1**  Diagnostic accuracy for (A) one FIT and (B) two FITs for advanced adenoma and inflammatory bowel disease | *page 2* |
| **Supplementary Table 2**  Local NHS Lothian population by socioeconomic deprivation | *Page 3* |
|  |  |

Supplementary Table 1: Diagnostic accuracy for (A) one FIT and (B) two FITs for advanced adenoma and inflammatory bowel disease

(A) Single FIT

|  | **AA** | | **IBD** | |
| --- | --- | --- | --- | --- |
|  | PRB | No PRB | PRB | No PRB |
| Sensitivity | 59.6% (50.5% – 68.2%) | 46.8% (38.3% – 55.5%) | 93.8% (86.2% – 97.3%) | 57.6% (39.2% – 74.5%) |
| Specificity | 66.7% (64.6% – 68.7%) | 82.4% (81.1% – 83.6%) | 67.6% (65.5% – 69.6%) | 81.7% (80.4% – 83.0%) |
| PPV | 9.2% (7.3% – 11.5%) | 8.9% (7.0% – 11.3%) | 10.1% (8.2% – 12.5%) | 2.9% (1.7% – 4.4%) |
| NPV | 96.7% (95.6% – 97.5%) | 97.7% (97.1% – 98.2%) | 99.6% (99.2% – 99.8%) | 99.5% (99.2% – 99.7%) |

(B) Two FITs

|  | **AA** | | **IBD** | |
| --- | --- | --- | --- | --- |
|  | PRB | No PRB | PRB | No PRB |
| Sensitivity | 76.6% (62.0% – 87.7%) | 60.0% (45.2% – 73.6%) | 100% (86.3% – 100%) | 62.5% (24.5% – 91.5%) |
| Specificity | 58.2% (55.0% – 61.4%) | 77.4% (75.3% – 79.4%) | 58.0% (54.8% – 61.2%) | 76.5% (74.3% – 78.5%) |
| PPV | 8.5% (6.0% – 11.5%) | 7.6% (5.2% – 10.7%) | 5.9% (3.8% – 8.5%) | 1.3% (0.4% – 2.9%) |
| NPV | 98.0% (96.5% – 99.0%) | 98.4% (97.6% – 99.0%) | 100% (99.3% – 100%) | 99.8% (99.3% – 99.9%) |

AA; Advanced adenoma, IBD; Inflammatory bowel disease, PPV: Positive predictive value, NPV; Negative predictive value, Two FITs; patients who have completed two FITs, maximum FIT result is used

Supplementary Table 2: Local NHS Lothian population by socioeconomic deprivation

| **SIMD** | **Proportion of NHS Lothian population** |
| --- | --- |
| 5 | 37.1% |
| 4 | 19.4% |
| 3 | 16.6% |
| 2 | 18.5% |
| 1 | 8.4% |

SIMD; Scottish Index of Multiple Deprivation (5, least deprived
